# On the spatial limits of parallel word processing in reading

**DOI:** 10.3758/s13414-025-03101-x

**Published:** 2025-06-05

**Authors:** Maša Mlinarič, Sander A. Los, Joshua Snell

**Affiliations:** Department of Experimental and Applied Psychology, Vrije Universiteit Amsterdam, Van der Boechorststraat 7, 1081 HV Amsterdam, The Netherlands

**Keywords:** Reading, Word recognition, Attention

## Abstract

Various models of reading assume that information from up to five words is processed in parallel. Although there is evidence that foveal words can be processed simultaneously with directly adjacent words, it remains to be seen whether three words is the limit. To empirically test this, we designed a lexical decision flanker task with three flankers on each side of the target. In two experiments (offline (N = 49) and online (N = 98)), target words were either orthographically unrelated to all flankers or repeated in one out of six flanker positions. Stimuli were briefly presented, allowing us to assume that flanker effects, if any, would stem from simultaneous rather than sequential processing of the target and flankers. We observed flanker effects for flankers immediately adjacent to the target word. However, the relatedness of flankers in more remote positions did not impact recognition of the target. Our results suggest that word processing occurs for approximately three words in parallel, which is more than what some theories (e.g., E-Z Reader, Chinese Reading Model) suggest, but less than what some other theories (e.g., Glenmore, OB1-reader) suggest.

## Introduction

According to various models of reading, multiple words are processed in parallel (Engbert et al., 2005; Reilly & Radach, 2006; Snell, 2024b; Snell et al., 2018c). The scope of this parallel processing has been taken to be approximately five words in OB1-reader, meaning that it processes information from up to five words simultaneously (Lopes-Rego et al., 2024; Snell et al., 2018c). While the parallel-processing assumption offers a strong theoretical basis for explaining several phenomena (see below), the spatial limits of parallel word processing have not yet been empirically established. The present study addresses this research gap.

Within the context of models like OB1-reader, SWIFT, Glenmore and PONG (Engbert et al., 2005; Reilly & Radach, 2006; Snell, 2024b; Snell et al., 2018c) the spatial limits of parallel word processing very much pertain to the scope of visuo-spatial attention that is assumed by those models. Therefore, we must first specify what we mean by visuo-spatial attention, and how this differs from other processing spans that have been conceptualized in the literature. Foremost, a distinction must be made between the attentional distribution and the so-called *perceptual span*, initially defined as the portion of text from which information can be gathered during a single fixation (McConkie & Rayner, 1975). Previous research has investigated the size of the perceptual span using the moving window technique, where a pre-determined number of characters left and right of the fixation point is shown as in the original text, while characters beyond those set boundaries are modified; for example, all letters are changed to Xs (McConkie & Rayner, 1975; McConkie & Rayner, 1976). This window within which text can be seen normally always moves along with the fixation point, and the smallest window whereby reading is unhampered relative to normal reading is taken to reflect the perceptual span. It was suggested that visual information is processed up to four character positions to the left and up to 12–15 to the right of the current gaze position (McConkie & Rayner, 1975; McConkie & Rayner, 1976; see also Jordan et al., 2016, for a more recent analysis).

The perceptual span can also be studied by investigating parafoveal preview effects using the gaze-contingent boundary paradigm (Rayner, 1975). Parafoveal preview effects indicate that readers are able to extract information from words in the parafovea, and use it when they come to fixate on those words later. In studies that use the gaze-contingent boundary paradigm, researchers place an invisible boundary in the text, and manipulate properties of a word after the boundary. While fixating before the boundary, the sentence is seen in its manipulated form, but when the gaze crosses the boundary, the manipulated word is replaced by the target word. There are numerous studies that set the boundary after word *n* and manipulated word *n + 1*, word *n + 2*, or both (e.g., Angele et al., 2008; Angele et al., 2013; Angele & Rayner, 2011; Dare & Shillcock, 2013; Kliegl et al., 2007; Rayner et al., 2007; Risse & Kliegl, 2012)*.* While parafoveal preview benefit effects on word *n + 1* have been widely reported (e.g., Angele et al., 2008, 2013; Angele & Rayner, 2011; Dare & Shillcock, 2013; Rayner et al., 2007), the existence of parafoveal preview benefit effects on word *n + 2* is still uncertain, with some studies failing to find it (e.g., Angele et al., 2008; Rayner et al., 2007), but also reports that suggest an effect in specific conditions (Angele & Rayner, 2011; Risse & Kliegl, 2012). Thus, taken together, parafoveal preview effects suggest that the perceptual span extends at least one word, and possibly two words, to the right of fixation.

We should emphasize that the aforementioned characteristics of the perceptual span are not entirely rigid. Indeed, the perceptual span is, to some extent, flexible, and varies depending on text difficulty (Henderson & Ferreira, 1990) and direction of reading (Pollatsek et al., 1981). Furthermore, the ranges mentioned above may reflect eccentricities at which there is the earliest visual disturbance, while actual word processing might be confined to a narrower range (this narrower window has fittingly been named the word identification span (Rayner, 1998; Rayner et al., 1982; Underwood & McConkie, 1985; also see Frey & Bosse, 2018, for a review of yet a few other processing spans)).

While the gaze-contingent boundary paradigm (Rayner, 1975) and the moving window technique (McConkie & Rayner, 1975, 1976) have led to the understanding that information can be obtained from parafoveal words, it cannot be concluded that readers gather all this information simultaneously – i.e., it does not evidence that attention is distributed across all words at once, as has been assumed by models like SWIFT (Engbert et al., 2005), Glenmore (Reilly & Radach, 2006), PONG (Snell, 2024b), and OB1-reader (Snell et al., 2018c). Alternatively, readers might firstly focus on the foveal word and covertly move their attention rightward after it has been recognized, before the fixation has ended. In other words, a wide perceptual span (encompassing multiple words) may be effectuated by even a narrow attentional distribution (encompassing one word at a time). In contrast, in the aforementioned models, visuo-spatial attention is conceptualized as a single gradient that continuously encompasses multiple words; this allocation of attention engenders orthographic processing across multiple words in parallel – whether strongly (at the gradient’s centre) or fleetingly (at the gradient’s tails). These models are thus best tested by studying parallel word processing, rather than parafoveal processing per se.

Parallel processing in reading can be studied by investigating parafoveal-on-foveal (PoF) effects in both the gaze-contingent boundary paradigm (Rayner, 1975) and the flanker task (pioneered by Eriksen & Eriksen, 1974). The former, contrary to studying the preview benefit effect, focuses on the effect of the previewed postboundary word on the preboundary word (Dare & Shillcock, 2013). Orthographic PoF effects can be studied by presenting a repetition of the preboundary word as a preview in the postboundary position, before it changes to the actual word after the gaze crosses the invisible boundary. The typical finding is that fixations on the preboundary word are shorter when the postboundary word is orthographically related to it, as compared to an unrelated postboundary word – a finding that has been replicated multiple times (e.g., Angele et al., 2013; Dare & Shillcock, 2013; Snell et al., 2017).

Given that the boundary paradigm already robustly suggests that attention is distributed across foveal and upcoming words simultaneously, what might be the added benefit of the flanker paradigm? While the former offers valuable insight into the scope of attention in reading in a more naturalistic setting, a potential caveat is that there is no control over the presentation duration of stimuli. If, say, the preboundary word is fixated for 300 ms, there would in principle be time for covert attention to shift back and forth between the pre- and postboundary word, potentially driving PoF effects even if attention was to encompass only one word at a time. This problem can be avoided in a flanker task, where the duration of the stimulus presentation can be tightly controlled.

When using a flanker task to assess the spatial limits of parallel word reading, its classic implementation, where stimuli are presented on the screen until participants respond, is not sufficient, because of the reasons outlined above (see also Snell & Grainger, 2018). On the contrary, stimuli need to be presented only briefly to ensure that any processing of the flankers must have coincided with processing of the target. Such a flanker task was first implemented by Dare and Shillcock (2013). They used 150-ms stimulus presentation durations and found that related orthographic information in the parafovea facilitates lexical decisions about the foveated word relative to unrelated parafoveal information, implying that readers can integrate information across multiple words. Numerous other studies have replicated this finding (e.g., Grainger et al., 2014; Snell et al., 2017; Snell & Grainger, 2018; Snell et al., 2018a, Snell et al., 2018b, Snell et al., 2019). It is worth mentioning, however, that as those studies did not mask the stimuli after they disappeared from the screen, it would hypothetically be possible to serially switch attention among representations in sensory memory (e.g., White et al., 2019). In line with more recent implementations of the flanker task in our lab (Snell, 2024a; Snell & Simon, 2025), we avoided this issue by using masks in our study.

Although some researchers have previously assumed that orthographic PoF effects may occur without the involvement of attention (e.g., Angele et al., 2013), recent evidence suggests that PoF effects in fact are driven by attention (Snell et al., 2018b). Snell, Mathôt, et al. built on the results of Mathôt et al. (2016), who suggested that covert attention can be tracked using pupillary light responses to covertly attended stimuli of different brightnesses. Snell, Mathôt, et al. performed pupillometry while participants were doing a flanker task with either vertically or horizontally aligned flankers. By manipulating the brightness of the flankers, they observed that the pupils only reacted to the brightness of horizontally aligned flankers. Concurrently, PoF effects were also only present for these sets of flankers, leading to the conclusion that they are driven by attention.^1^ This is nicely in line with the OB1-reader, Glenmore, PONG and SWIFT models, according to which ‘to attend’ inevitably implies ‘to orthographically process’ (Engbert et al., 2005; Reilly & Radach, 2006; Snell, 2024b; Snell et al., 2018c).

But at the same time these models do not tell the whole story. While we argue that the PoF effect indicates that a word must have been attended, the absence of a PoF effect does not indicate that it was not attended. It could be that attention was indeed administered to a certain word, but that other constraints, such as acuity and crowding, prevented any potential PoF effects. In this light, our results will outline the window across which orthographic information is integrated and processed in parallel (knowing that attention was administered to words in this window), while no conclusions could be made about whether this window captures the entirety of the attentional distribution.

At any rate, since the previously mentioned studies investigating PoF effects manipulated the orthographic relatedness of parafoveal words or bigrams directly next to the target (Dare & Shillcock, 2013; Grainger et al., 2014; Snell et al., 2017; Snell & Grainger, 2018; Snell et al., 2018b), it seems safe to say that parallel processing proceeds at least for three words, while leaving unanswered the question of whether three is the limit. It could be that words further away from the central one are included in the window of parallel processing (as is assumed by Glenmore (Reilly & Radach, 2006) and OB1-reader (Snell et al., 2018c)), but this cannot be investigated when the stimulus consists of two or three strings of letters overall (one foveal target word and one or two flankers). Hence, we decided to use a more elaborate version of the flanker task in order to study the spatial limits of parallel word processing in greater detail.

We decided to expand the flanker paradigm by using multiple flankers on each side, manipulating each flanker individually to be able to investigate whether distant words can be processed simultaneously with the central word. While this, to our knowledge, constitutes a first-ever test of the scope of parallel word processing beyond three words of this kind (i.e., a target word and two adjacent flankers), it is worth mentioning one related study of Fournet et al. (2023). They employed a flanker task with multiple bigrams on each side of the target word, manipulating each set of bigram pairs (inner, outer) independently. They found that when repeating both sets of related bigrams (RO RO ROCK CK CK), facilitation did not significantly differ from trials with repeated related inner bigrams and unrelated outer bigrams (CA RO ROCK CK SH). They proposed that only the target word and its adjacent flankers are included in the process of spatial integration of orthographic information. They further hypothesized that while non-adjacent flankers do not partake in this process, their presence does influence the scope of attention by broadening it. This would then lessen the PoF effects of adjacent flankers. Supporting their hypothesis, they found that single repeated bigrams (RO ROCK CK) facilitated word recognition more when compared to two sets of related flankers (RO RO ROCK CK CK); and while they did not find a significant main effect of the number of flankers, there was nonetheless an interaction between flanker relatedness and number of flankers. Still, the fact that the orthographic relatedness of outer bigram flankers did not affect performance could be taken to suggest that the window of orthographic processing is quite confined, extending only a few letters beyond the word in focus. Arguably, however, the story is not complete: their study only included the simultaneous manipulation of each set of bigram pairs (inner, outer), and could, therefore, not be used to reveal any asymmetries in the attentional distribution (see, e.g., Snell & Grainger, 2018). Additionally, bigram flankers do not resemble normal text, and therefore have less ecological validity than whole-word flankers.

In sum, various models of reading (OB1-reader, Glenmore, SWIFT, PONG) assume that attention encompasses multiple words, and that this engenders parallel orthographic processing across those words (Engbert et al., 2005; Reilly & Radach, 2006; Snell, 2024b; Snell et al., 2018c); however, these assumptions have not yet been empirically tested. To address this issue, we designed a study with three flanking words on each side of the target word and manipulated each of the flanker positions individually, to investigate the spatial limits of parallel word reading. We hypothesized this window to encompass words to both the left and the right from the fixation with a rightward bias. Meanwhile, we are aware that potential effects of more remote flankers may be blocked by influences unrelated to attention, such as crowding and acuity (Bouma, 1970; Legge et al., 2001; Pelli et al., 2007; Veldre et al., 2023; Yeshurun & Rashal, 2010). We address these constraints further in the *General discussion*.

## Experiment 1

### Method

#### Participants

Results from 49 people (average age 20.7 years) were collected. All of the participants self-reported to be non-dyslexic readers with normal or corrected-to-normal vision and to have Dutch as their first language. They all provided signed informed consent prior to the start of the experiment and were given either course credits or €7.50 in exchange for participation. The study was approved by the Ethics committee of the Faculty of Behavioural and Movement Sciences of the Vrije Universiteit Amsterdam.

#### Stimuli

We used the Dutch Lexicon Project (DLP) database (Keuleers et al., 2010) to identify 420 four-letter words and grouped them into 210 pairs which consisted of a target word and a flanker word. Targets and flankers were semantically unrelated and had no letters in common. Similarly, we retrieved 420 four-letter non-words from the DLP database, paired into 210 target non-words and non-word flankers. In that way, a stimulus either consisted solely of words or non-words. Flanker and target words were selected and matched by their frequencies by the following procedure. The frequency of each candidate word was calculated by averaging CELEX frequency and SUBTLEX frequency (both raw frequencies) provided by the DLP database to get a list of the 600 most frequent four-letter words. One word containing an apostrophe and one inappropriate word were removed. Using this initial list, we created our 210 pairs of words, matching them by their average frequency. The average frequency of all targets and all unrelated flankers was virtually equal at 89.57 parts per million (ppm) for targets and 89.93 ppm for unrelated flankers.

#### Apparatus

The experiment was created using OpenSesame (Mathôt et al., 2012). Participants were presented with the instructions for the experiment on-screen and also had them explained by the investigator prior to the start of the experiment. Participants were seated in a dimly lit room, 80 cm away from the screen,^2^ so that each character space took approximately 0.23° of visual angle (screen characteristics: screen diagonal: 23.8 inches, resolution: 1,920 x 1,080 pixels, refresh rate: 240 Hz).

#### Design

**Fig. 1:**
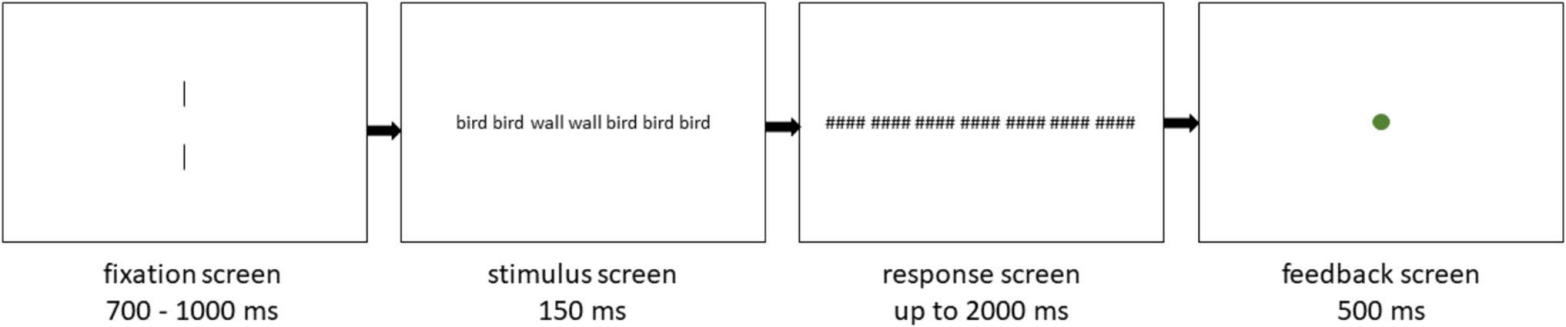
Visual representation of a single trial with the target word ‘wall’ and the flanker word ‘bird’ in the *n – 1 related* flanker condition. Proportions in the figure are inaccurate as words are enlarged for better visibility

Each target word appeared with its designated flanker in seven different conditions, as illustrated in Table 1 (note that a single trial is visualised in Fig. 1). In the *unrelated* flanker condition, the target word was flanked on both sides by three instances of its designated, unrelated flanker word. In the remaining six *related* flanker conditions, one of the six flankers was a repetition of the target word, while the remaining flankers were unrelated to the target. These six related flanker conditions were labelled *n* *– 3* to *n + 3*, referring to the location of the related flanker relative to target location *n* (note that this labelling is analogous to that used in boundary paradigm studies).

**Table 1:** Representation of all the flanker conditions used in the study

Flanker condition	Flanker *n –* *3*	Flanker *n –* *2*	Flanker *n –* *1*	Target	Flanker *n + 1*	Flanker *n + 2*	Flanker *n + 3*
*unrelated*	bird	bird	bird	wall	bird	bird	bird
*n – 3 related*	wall	bird	bird	wall	bird	bird	bird
*n – 2 related*	bird	wall	bird	wall	bird	bird	bird
*n – 1 related*	bird	bird	wall	wall	bird	bird	bird
*n + 1 related*	bird	bird	bird	wall	wall	bird	bird
*n + 2 related*	bird	bird	bird	wall	bird	wall	bird
*n + 3 related*	bird	bird	bird	wall	bird	bird	wall

*Note:* Visual representation of all of the seven flanker conditions on an example with the target word ‘wall’ and the flanker word ‘bird’. The same flanker conditions were implemented for non-word trials

Using a Latin square design, we divided participants in seven groups, ensuring that all words were shown in all seven conditions, but each word only once per person, amounting to 30 trials per condition per participant. With 1,470 trials per condition, we approached the recommended value of 1,600 proposed by Brysbaert and Stevens (2018) to reach a statistical power of ~ 0.80. Statistical power of 0.96 was calculated based on 2,000 measurements per condition from the orthographic flanker task data of Snell and Grainger (2018), indicating that orthographic effects are robust (Snell et al., 2021). Therefore, we believe that we reached a sufficient statistical power in spite of the slightly lower number of trials per condition. Trials with non-words were manipulated in the same fashion. All trials appeared in a randomised order. Non-words were implemented exclusively to induce the lexical decision task. Consequently, the data derived from those trials were not the focus of this paper.

The experiment started with an unlogged practice run containing 28 trials and continued with the logged, official run. The latter contained 420 trials, amounting to a total duration of approximately 20 min. The stimuli from the practice run were repeated in the official run. Participants were offered a break after the first 210 trials of the logged part of the experiment. The experimental procedure is illustrated in Fig. 1. Each trial started with a fixation display with vertical bars at the centre of the screen. Participants were instructed to fixate their gaze between those vertical bars. The duration of the fixation display was random, varying from 700 ms to 1,000 ms in order to avoid automatic, rhythmic button-pressing at the cost of accuracy. The fixation screen was followed by a brief presentation of the stimulus. The target and its flankers were presented simultaneously at the centre of the screen in one of the 14 different conditions (flanker x lexical status). Flankers and target words were separated with a single space. After 150 ms, the stimulus was replaced by masks. Participants were instructed to decide whether the target was a word or a non-word, indicating their decision with the press of the keyboard button (M or C on the QWERTY keyboard), as quickly and accurately as possible. The same lexicality category (word or non-word) was shown a maximum of four times in a row. If no response was given after 2,000 ms, the trial was marked as incorrect. Each trial was followed with a 500-ms feedback screen that presented a green or red dot at the centre of the display, representing a right or a wrong answer, respectively. Participants were also shown their average response time (RT) and accuracy at the end of each block of the experiment (practice run, first half and second half).

### Results

With 49 participants, there were 1,470 word trials per condition, amounting to a total of 10,290 word trials. Non-word trials were not included in the analysis. All participants reached sufficient accuracy (≥ 70%). Incorrectly answered word trials (10.36%) were not included in the analysis of RTs. We calculated average RTs and standard deviations (SDs) per participant per flanker condition and excluded word trials that were more than 2.5 SDs away from the mean (2.83% of the remaining trials). Consequently, 8,963 word trials were used for the analysis of RTs. When analysing accuracies, we applied the same upper and lower cut-off boundaries per condition per participant to exclude outliers for the incorrectly answered word trials as well, amounting to a total of 9,895 trials.^3^

We analysed data using linear mixed-effects models (LMMs) and generalized linear mixed-effects models in the R Statistical Software (R Core Team, 2023) with flanker condition as a fixed effect and participants as random effects (Baayen et al., 2008). We started with a model with maximal random structure. Due to singularity issues with a model with both random slope and intercept, we used a simpler model with by-participant random intercept for the analysis of RTs. As the most complex model did not converge for the analysis of accuracies, we used a model with by-participant random intercept only. The model for the analysis of RTs was fitted with the lmer function, and the one for the analysis of accuracies with the glmer function from the lme4 package (Bates et al., 2015). We calculated *t* values (and *z* values for the analysis of accuracies), regression coefficients (*b* values), and standard errors (SEs). Results were interpreted as significant when *t* > |1.96| (and *z* > |1.96| for the analysis of accuracies), which corresponds to α = 0.05. The *unrelated* flanker condition was compared to all the other related flanker conditions. As the RTs were not normally distributed, we log-transformed the outliers-free RT data for the LMM analysis.

#### Response times

**Fig. 2:**
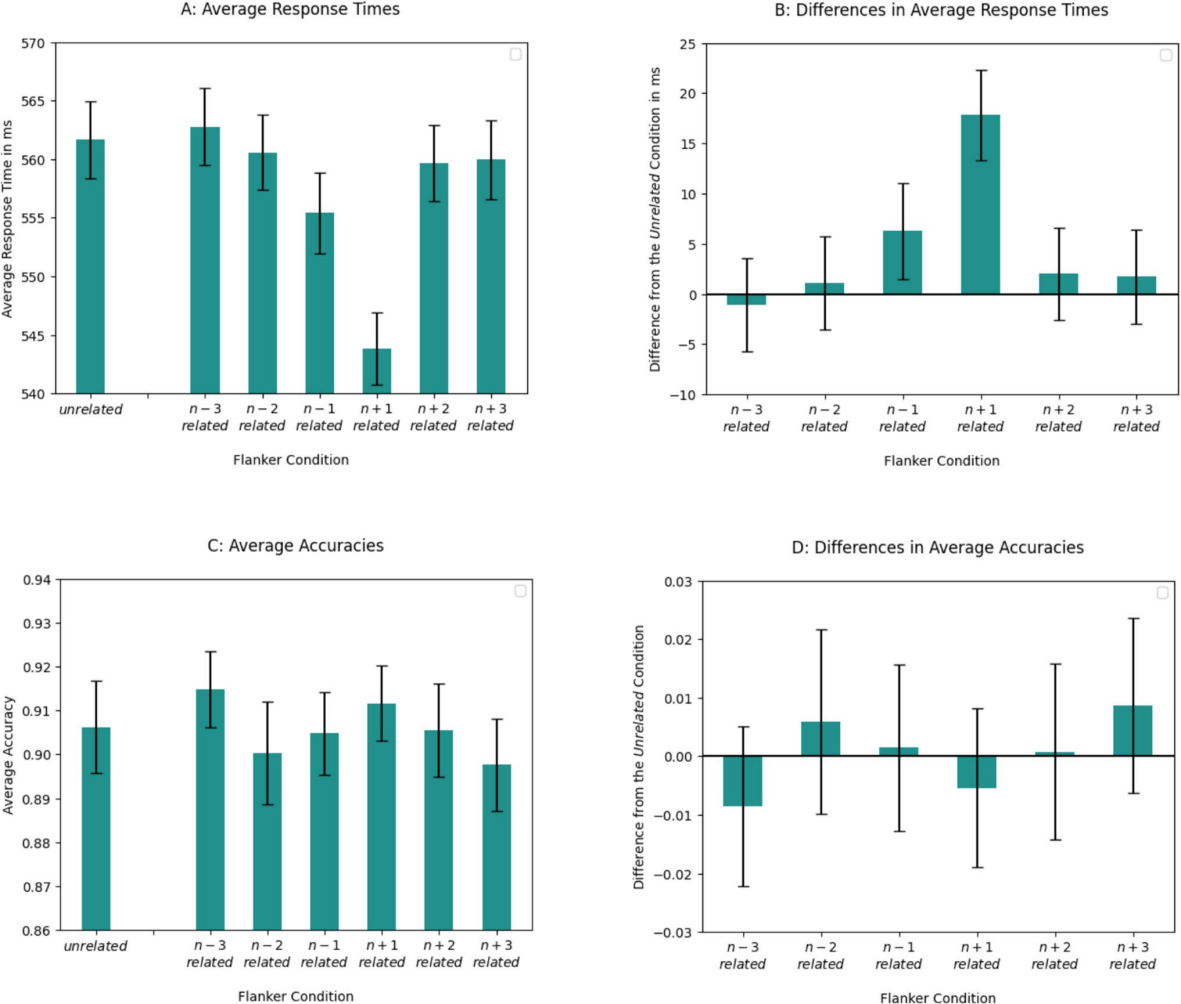
Results of average response times and accuracies per condition for Experiment 1. Though we used log-transformed response times (RTs) in the analyses, we plotted non-transformed RT data on the graphs for easier understanding. **A:** Average RTs (in ms) per condition. Error bars represent SEs that were calculated from the non-transformed RTs. **B:** Difference (in ms) in average RTs between the unrelated condition and other conditions. Values were obtained by subtracting average RT of a given condition from the average RT of the unrelated flanker condition. Error bars represent SEs of a difference. **C:** Average accuracies. Error bars represent SEs. **D:** Difference in average accuracies between the unrelated condition and other conditions. Error bars represent SEs of a difference

Average RTs and difference scores for average RTs in the *unrelated* condition versus other conditions are presented in Fig. 2. RTs in both the *n – 1* and the *n + 1* *related* flanker conditions were significantly shorter than RTs in the *unrelated* condition (see Table 2). Furthermore, RTs in the *n +* *1 related* flanker condition were significantly shorter than in the *n – 1* *related* flanker condition (b = −0.02, SE = 0.01, t = −2.23). Lexical decision was not significantly facilitated in any other flanker condition.

**Table 2:** The analysis of response times (RTs) and accuracies for Experiment 1, with the unrelated flankers condition as reference

Flanker condition	Experiment 1					
	RTs			Accuracies		
	*b*	*SE*	*t*	*b*	*SE*	*z*
*n – 3 related*	−0.11*10^−2^	0.68*10^−2^	−0.16	0.10	0.13	0.78
*n – 2 related*	−0.31*10^−2^	0.68*10^−2^	−0.45	−0.07	0.13	−0.56
*n – 1 related*	−1.55*10^−2^	0.68*10^−2^	**−2.27**	−0.02	0.13	−0.16
*n + 1 related*	−3.08*10^−2^	0.68*10^−2^	**−4.50**	0.06	0.13	0.45
*n + 2 related*	−0.40*10^−2^	0.68*10^−2^	−0.59	−0.01	0.13	−0.07
*n + 3 related*	−0.50*10^−2^	0.68*10^−2^	−0.73	−0.10	0.13	−0.76

*Note:* Significant results are given in bold

*SE* standard error

#### Accuracies

The results are presented in Fig. 2. There was no significant effect of the flanker condition on the accuracies (see Table 2).

## Experiment 2

### Method

Experiment 2 was an online version of Experiment 1. The experiments were identical and used the same stimulus list. In Experiment 2, the constraint on the number of repetitions of the same target category in a row was omitted. We uploaded our experiment to JATOS server (Lange et al., 2015) and chose Prolific (www.prolific.com) as the platform to recruit participants. We set our pre-screening criteria as follows: non-dyslexic readers with normal or corrected-to-normal vision and Dutch as their first language. Additional criteria for our online experiment were fluency in English, a Prolific approval rate above 94% and age between 18 and 35 years. While fluency in English was required to ensure a good understanding of our Consent Form, Participant Information Form and instructions, the other criteria were implemented to get dedicated participants who were age-matched (average age 26.7 years) to our in-person participants. Participants received a monetary compensation of £5.50.

Participants were asked to only use a laptop or desktop computer. They provided their consent and validated their pre-screening answers prior to the start of the experiment. If they did not consent or indicated that any of the exclusion criteria applied to them, the experiment ended with the request to return their submission. They were able to stop the experiment at any time, which would result in an incomplete dataset that was not used for the analysis.

In Experiment 2 we implemented counterbalancing by randomly assigning people to one out of seven groups at the beginning of the experiment. Only people who submitted their complete dataset (exactly 420 trials) on their first run of the experiment were included in the analysis. Prior to the start of the study, we decided to stop the data collection once 14 unique individuals with a sufficient overall accuracy (≥ 70%) were in each group, amounting to a total of 98 participants (double the size of our in-person experiment, to compensate for the increased amount of statistical noise that was anticipated with online experimentation). With 2,940 trials per condition, we surpassed the recommended 1,600 trials per condition to reach a statistical power of ~ 0.80 (Brysbaert & Stevens, 2018).

### Results

We collected a total of 20,580 word trials, amounting to 2,940 word trials per condition. Following the same procedure as in Experiment 1, we excluded 9.57% incorrectly answered word trials. From the remaining trials, an additional 2.57% were excluded due to being more than 2.5 SDs away from the specific person’s flanker condition’s mean. The remaining 18,132 word trials were used for RT analysis. We used the same cut-off boundaries per condition per participant to exclude outliers for the analysis of accuracies, amounting to a total of 19,872 word trials.^4^

We used R Statistical Software (R Core Team, 2023) to run LMMs with flanker condition as a fixed effect and participants and items (targets) as random effects (Baayen et al., 2008). We started with a model with maximal random structure. Due to singularity issues with more complex models, we used a model with by-participant and by-target random intercept. The same model was used for the analysis of accuracies, as more complex models did not converge. The model for the analysis of RTs was fitted with the lmer function, and the one for the analysis of accuracies with the glmer function from the lme4 package (Bates et al., 2015). As the results were not normally distributed, we log-transformed outliers-free RTs for the LMM analysis. *Unrelated* flanker condition was set as a reference and compared to all the other *related* flanker conditions. We calculated *b* values, SEs, *t* values (and *z* values for the analysis of accuracies). Results were interpreted as significant when *t* > |1.96| (and *z* > |1.96| for the analysis of accuracies), which corresponds to α = 0.05.

#### Response times

**Fig. 3:**
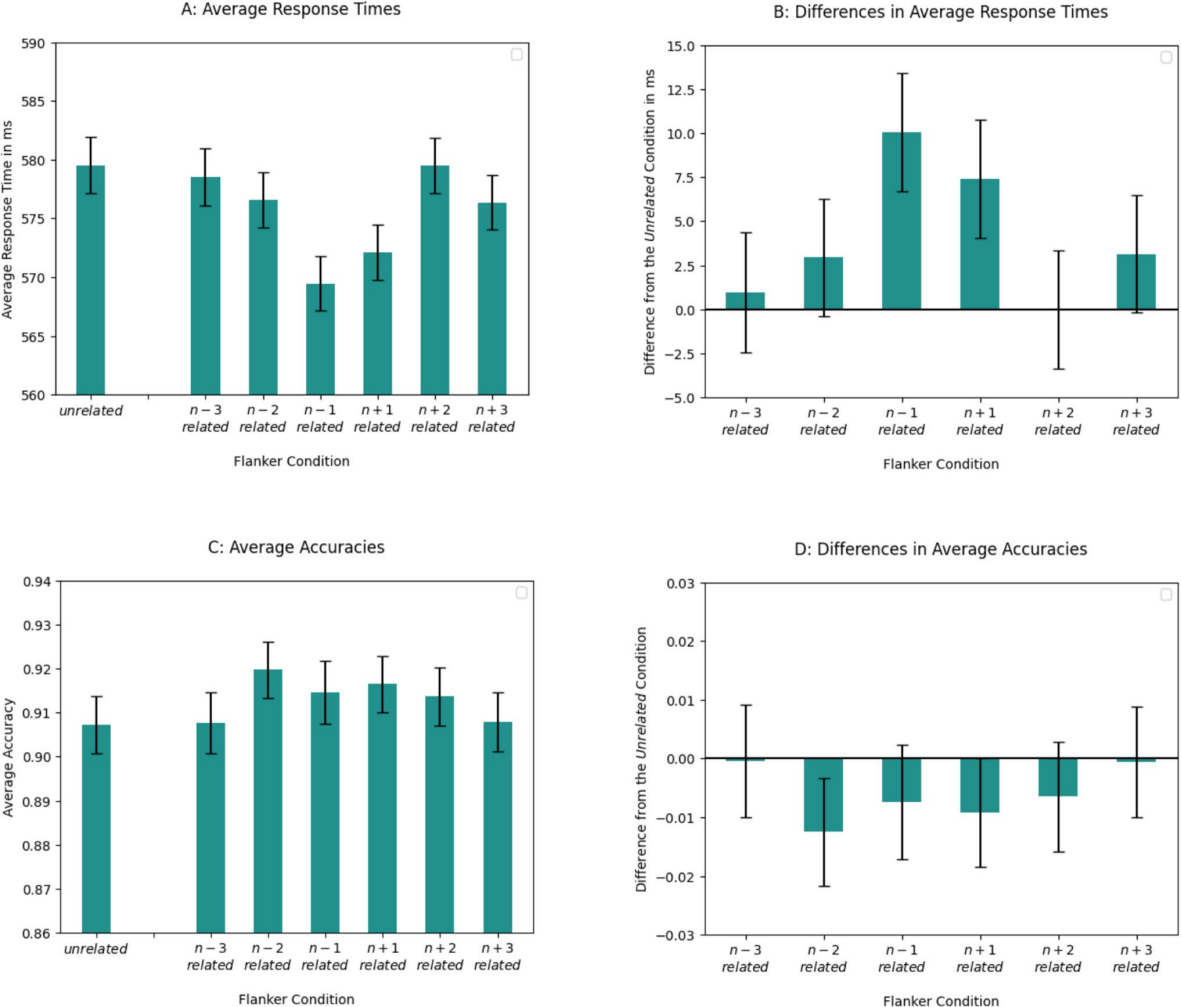
Results of average response times (RTs) and accuracies per condition for Experiment 2. Though we used log-transformed RTs in the analyses, we plotted non-transformed RT data on the graphs for easier understanding. **A:** Average RTs (in ms) per condition. Error bars represent SEs that were calculated from the non-transformed RTs. **B:** Difference (in ms) in average RTs between the unrelated condition and other conditions. Values were obtained by subtracting average RT of a given condition from the average RT of the unrelated flanker condition. Error bars represent SEs of a difference. **C:** Average accuracies. Error bars represent SEs. **D:** Difference in average accuracies between the unrelated condition and other conditions. Error bars represent SEs of a difference

Average RTs per condition and the difference in average RTs between the *unrelated* flanker condition and other flanker conditions are shown in Fig. 3. Repeated orthographic information one position left and right from the target word (i.e., the *n – 1* and *n + 1 related* flanker conditions*)* significantly facilitated lexical decisions. RTs in those two conditions did not significantly differ from each other (b = −0.39*10^−2^, SE = 0.44*10^−2^, t = −0.88). No PoF effects were observed for the flankers that were more distant from the target word.

#### Accuracies

The results are shown in Fig. 3. There was no significant effect of the flanker condition on the accuracies. Analysis for both RTs and accuracies can be found in Table 3.

**Table 3:** The analysis of response times (RTs) and accuracies for Experiment 2

Flanker condition	Experiment 2					
	RTs			Accuracies		
	b	SE	t	b	SE	z
*n – 3 related*	−0.33*10^−2^	0.44*10^−2^	−0.75	0.01	0.10	0.12
*n – 2 related*	−0.53*10^−2^	0.44*10^−2^	−1.22	0.16	0.10	1.64
*n – 1 related*	−1.80*10^−2^	0.44*10^−2^	**−4.10**	0.11	0.10	1.10
*n + 1 related*	−1.41*10^−2^	0.44*10^−2^	**−3.22**	0.14	0.10	1.39
*n + 2 related*	−0.11*10^−2^	0.44*10^−2^	−0.26	0.10	0.10	1.06
*n + 3 related*	−0.53*10^−2^	0.44*10^−2^	−1.21	0.01	0.10	0.08

*Note:* Significant results are given in bold

*SE* standard error

### Discussion

The aim of our study was to determine the spatial limits of parallel word processing, by using a modified version of a flanker task with three word flankers on each side of the target word. In doing so, we built on prior work showing that orthographic PoF effects – which have been robustly revealed in previous flanker studies – are driven by covert attention (Snell et al., 2018b). Our results suggest that the window of integration of orthographic information spans across approximately three words, which translates to approximately 3.24° of visual angle for our in-person experiment. Participants were able to recognize the target word significantly faster when its adjacent flanker on the left or right was a repetition (*n – 1* and *n + 1 related* flanker condition), compared to when all six flankers were unrelated. Meanwhile, there were no significant differences between the *unrelated* flanker condition and flanker conditions when the target was repeated in places *n – 3, n – 2, n + 2,* and* n + 3.*

Whether these spatial limits of parallel word processing also inform the size of other spans – such as the scope of visuo-spatial attention – depends on one’s theoretical stance. According to the OB1-reader (Lopes-Rego et al., 2024; Snell et al., 2018c), PONG (Snell, 2024b), Glenmore (Reilly & Radach, 2006) and SWIFT (Engbert et al., 2005) models, to reveal one is to reveal the other. That is, in line with these models we propose that visuo-spatial attention was at least administered to words in this window (leftward flanker, target word and rightward flanker). We can only speculate as to whether we have uncovered the limits of attention as such. It could be that while attention was administered to words in the farther periphery, no flanker effects were observed due to other limitations, such as acuity and crowding. With the most distant letters being approximately 3.93° from the centre, we assume that acuity itself was sufficient even for the outermost flankers. Here we refer to the seminal work of Bouma (1970), who investigated effects of eccentricity in a letter-recognition task. In his experiment, single letters without any flanking letters (i.e., no crowding) at an eccentricity of 4° were correctly recognized more than 80% of the time, suggesting acuity per se does not preclude orthographic processing at this eccentricity.^5^ In a more recent study, Cauchi and Meeter (in press) investigated flanker relatedness effects when flankers were placed at different eccentricities, and observed that flankers are processed at high eccentricities (4.29° from the fixated word).

In the aforementioned study, Bouma (1970) showed that the fraction of correctly identified letters does not only decrease with eccentricity, but also with increased crowding, caused by the addition of flanking letters on one or both sides of the target letter. We speculate, however, that crowding was not a key factor in our study either. McConkie and Rayner (1975) suggested that orthographic information is picked up and integrated up to at least ten character positions right from fixation (a result obtained by manipulating the confusability of letters outside the moving window). If crowding and acuity were dominating factors at these eccentricities, the window suggested by McConkie and Rayner would have been smaller. Since our *n + 2* flankers fell within this window, it is possible that crowding and acuity were not the primary reason for the lack of flanker effects in our study. Naturally, this is only speculation, as we did not test whether the second (and third) flanker on the left and right from the target word were actually legible to the participants. This may be an interesting idea for a follow-up study, wherein we would probe not only recognition of the central target stimulus, but also recognition of the flanking stimuli.

Assuming that crowding and acuity limits were not the key reason driving the lack of flanker effects from distant flankers in our study, we explore one last alternative possibility. While Fournet et al. (2023) proposed that spatial pooling of orthographic information in reading solely operates across the target word and its adjacent flankers, they also hypothesized that the addition of nonadjacent flankers nonetheless increases the spread of attention and consequently hinders target processing. If this were true, then attention may technically be distributed across more than three words. Indeed, although previous research suggests that orthographic PoF effects are driven by attention (Snell et al., 2018b), we should acknowledge that the area across which visuo-spatial attention is distributed may not necessarily perfectly overlap with the area from which orthographic information is integrated. Possibly orthographic processing at a given location only occurs if a certain amount of processing resources are allocated to that location. It may be that limited (negligible) amounts of attention might have leaked to peripheral stimuli that are not orthographically processed.

Importantly, there is tentative evidence for the above scenario. Snell and Grainger (2018) have argued that the placement of a flanker outside the scope of effective attention induces a partial shift of attention away from the attentional locus (i.e., attention is ‘smeared out’), consequently decreasing processing power for all words within the attentional span. As a consequence of this shift of attention, the impact from all flankers is reduced. This prompts two predictions: Firstly, overall RTs should be longer in our experiments compared to the experiments of Fournet et al. (2023) (since there were more flankers outside the scope of effective attention in our study); and secondly, our flanker effects should be smaller than those of Fournet et al. (2023). Both these predictions seem to hold: comparing their results from online experiments to RTs of our Experiment 2 (also an online experiment), we observe that RTs in our experiment are indeed longer. Additionally, the relatedness effect for adjacent flankers was larger in the study of Fournet et al. (2023), as in our study. As there are differences between the two experiments (e.g., the average frequency of their target words was slightly lower), it is of course important to acknowledge that the presented evidence is tentative.

Attention aside, the lack of effect for more distant flankers suggests that the window of parallel word reading is narrower than what is assumed by most aforementioned ‘parallel processing’ models (Reilly & Radach, 2006; Snell, 2024b; Snell et al., 2018c). At the same time these results refute serial word-processing models like E-Z Reader (Reichle et al., 1998) and the Chinese Reading Model (Li & Pollatsek, 2020). The rather provocative conclusion may thus be that *all* models were wrong, and that the truth instead lay somewhere in the middle, where parallel word processing is possible albeit within relatively strict spatial limits.

We wish to conclude with two methodological notes. Firstly, to what extent can an artificial task such as the flanker paradigm inform natural reading? In terms of visual input, it could be argued that our task, with three flankers on each side of a target word, was a better approximation of natural sentence reading than previous implementations of the flanker task (e.g., Dare & Shillcock, 2013; Grainger et al., 2014; Snell et al., 2017; Snell & Grainger, 2018). Our flanker effects (~ 13 ms average across both experiments) are indeed similar to what has been observed in sentence-reading studies that tested PoF effects from word *n + 1* on word *n* with the boundary paradigm (e.g., Snell et al., 2017). We nonetheless have to acknowledge that the flanker paradigm inevitably remains quite different from natural reading. The biggest difference is arguably that readers do not make eye movements in the flanker paradigm. The perceptual span is thought to be largely determined by readers’ desire to move their eyes (e.g., Rayner, 1998), and therefore one might predict, for instance, stronger effects from *n + 1* (and possibly an effect from *n + 2*) in sentence reading as compared to what we have reported here. We would expect the lack of effects from *n – 2* to replicate in a sentence reading study. At the same time the flanker paradigm yields important benefits. For one, it enables us to tightly control stimulus presentation times, thereby giving more certainty to inferences about the part of the text that can be processed simultaneously. This is harder to achieve in a naturalistic setting, where words are presented on the screen indefinitely. At any rate, it seems that even when instructed to do so, participants were unable to limit their attention to a single word*.* If this is not possible in a simple lexical decision flanker task, we speculate it may well not be possible in a naturalistic setting, where it is probably often beneficial to extend attention to the right, in the direction of reading. With this in mind, we believe that we have at least uncovered the smallest possible window of parallel processing.

On a second methodological note, we may ask ourselves whether online experiments are a useful tool when answering questions about the attentional distribution in reading. Comparing the results from the two experiments, we see that while we observed PoF effects from the leftward and rightward flankers in both experiments, the rightward attentional skewness was only present in Experiment 1. Upon further investigation, it seems that it was not driven by all of the readers, but by a substantial bias of those who do exhibit it.^6^ In short, as both experiments offered similar results in terms of the spatial limits of parallel word processing, we argue that online experiments may be a reliable source of data depending on the question at hand.

What do our findings imply for future work in the field of reading research? The results of our study suggest that the integration of orthographic information operates across approximately three words, which translated to approximately 3.24° of visual angle for our in-person experiment. Additional research with different word lengths for both flankers and targets is needed to test the robustness of that number and to determine whether these spatial limits are best expressed in terms of number of words or number of letters. In the future, this could be done by using a multi-word flanker task with five-letter or six-letter words. A more precise approach would be to use a continuous, long string of letters on each side of the target, and manipulate orthographic relatedness at the level of single characters. This would allow for a more fine-grained delineation of the gradient shape of attention. Another possible line of experiments could investigate whether the reading brain can orthographically process nonadjacent words in specific conditions (e.g., when encountering shorter stimuli). Lastly, though we believe that acuity to detect nonadjacent flankers in our study was sufficient, one could conduct a follow-up experiment to verify that the lack of attention on those flankers (and not their decreased acuity), was really the reason why there were no PoF effects. One could make adjacent flankers more blurry to match their acuity with the nonadjacent flankers. If PoF effects from adjacent flankers were observed in this more blurry condition, it could be concluded that the acuity to detect nonadjacent flankers in our original experiment was indeed sufficient.

In conclusion, based on the results of our two experiments, we suggest that the spatial limits of parallel processing in reading are narrower than assumed by OB1-reader (Snell et al., 2018c), PONG (Snell, 2024b) and Glenmore (Reilly & Radach, 2006), but broader than assumed by E-Z Reader (Reichle et al., 1998) and the Chinese Reading Model (Li & Pollatsek, 2020), with parallel processing operating within a span of approximately three words.

## Data Availability

The data and materials for both experiments are available via the Open Science Framework (OSF) (https://osf.io/uv3y5/). None of the experiments were preregistered.

## Footnotes

1 We believe that the added value of this study is not in showing that people attend to horizontal, and not vertical, flankers, but that attention can be inferred via flanker effects.

2 This is an approximate distance as no chinrest was used.

3 Data for non-words were analysed using the same procedure as for word trials. While there were no significant effects in the analysis of RTs, participants were less accurate in the *n + 1 related* flanker condition compared to the *unrelated* condition (b = -0.25, SE = 0.12, z = -2.02). There were no other significant differences in the analysis of accuracies.

4 For non-words, there were again no significant differences between the related and the unrelated condition in RTs. Participants were significantly less accurate in the *n + 1 related* flanker condition than in the *unrelated* condition (b = -0.21, SE = 0.10, z = -2.16) and in the *n + 2 related* (b = -0.19, SE = 0.10, z = -1.96) flanker condition than in the *unrelated* condition.

5 Our stimuli are comparable in size; though our viewing distance was longer, we used appropriately larger font.

6 While 28 people in Experiment 1 showed a shorter mean RT in the *n + 1 related* flanker condition compared to mean RT in the *n – 1 related* flanker condition, the direction was reversed for the remaining 21 people Individual’s difference was calculated as (mean RT in the *n + 1 related* condition) minus (mean RT in the *n – 1 related* condition). It was either a negative or a positive value. The difference between the sum of all positive values and the sum of all negative values was 509 ms (in the direction of negative values – lower RT for the *n + 1 unrelated* condition). In Experiment 2, 48 participants exhibited shorter mean RTs in the *n + 1 related* condition, while 50 did not. There was a difference of 236 ms (*n + 1 related* flanker condition elicited longer RTs: note that while the number of people was calculated using the log-transformed data, the difference in ms was calculated using the non-log transformed data for better illustration).

## Code availability

The codes for both experiments are available via the Open Science Framework (OSF) (https://osf.io/uv3y5/).
